# COVID-19 and haematology services in a cancer centre from a middle-income country: adapting service delivery, balancing the known and unknown during the pandemic

**DOI:** 10.3332/ecancer.2020.1110

**Published:** 2020-09-24

**Authors:** Vivek S Radhakrishnan, Reghu K Sukumaran Nair, Gaurav Goel, Venkatraman Ramanan, Mammen Chandy, Reena Nair

**Affiliations:** 1 Division of Clinical Haematology Oncology and Hematopoietic Cell Transplantation, Tata Medical Center, Kolkata 700160, India; 2 Paediatric Haematology-Oncology, Tata Medical Center, Kolkata 700160, India; 3 Clinical Microbiology and Infection Control, Tata Medical Center, Kolkata 700160, India; 4 Executive Office, Administration, Tata Medical Center, Kolkata 700160, India; a https://orcid.org/0000-0001-9484-5669

**Keywords:** COVID-19, haematology services, cancer centre, middle-income

## Abstract

The COVID-19 pandemic has caused major disruptions in multiple spheres of healthcare delivery in the world. Developing nations have had to tackle this unanticipated crisis in the midst of various other healthcare delivery issues and resource constraints. As a tertiary level cancer care provider located in an eastern Indian city, a COVID-19 hotspot, we share our experience from the perspective of haematology and haematopoietic stem cell transplantation (HSCT) services. The primary challenges related to infection control included infection screening and decreasing exposure among patients and healthcare workers. Logistic challenges include maintaining essential patient care services, personnel redeployment, blood bank inventory constraints and maintaining the supply chain for a continuum of care. Clinical management challenges were dealt with by rationalising treatment delivery by modification of treatment regimens, risk-based deferral of HSCT, management of COVID-19 in patients, and staggering the follow-up schedules in survivors and those on maintenance therapies, among other strategies. These challenges were compounded by the restrictions imposed by a countrywide lockdown in the initial period of the pandemic, which also affected the socio-economic aspects of treatment delivery. As a training institution, this period also impacted academics and research activities. This overview details our response to these challenges during the COVID-19 pandemic, which has many unknowns.

The COVID-19 pandemic has been an unprecedented global crisis with far reaching socio-economic and healthcare disruptions. After the World Health Organization declared SARS-CoV-2 infection to be a pandemic on 11 March 2020, the immediate impact of the pandemic was on healthcare services [1]. The complexities of modern healthcare delivery and specialisation have made it even more difficult to quickly adapt to the rapidly changing scenario created by the pandemic. India has been one of the countries which had rapidly escalated the response to the pandemic with a country wide lockdown aimed at curtailing community transmission and build capacity [2]. The surge in cases from zero to over 1,700 and 171 reported deaths occurred in 4 weeks, and at the time of writing this paper has risen to 1,038,716 confirmed cases (and 26,273 total deaths) [3]. The initial phase of testing was highly selective for those with history of travel, close contact or significant disease burden. A mortality rate of around 10% was recorded initially and with the widespread availability of testing, it is about 2.5% at the time of manuscript preparation [3].

Cancer care is one of the most critical specialties where tightly controlled protocol-based therapy (and sometimes intensive treatments) and its timely delivery with active supportive care are essential to deliver good outcomes. COVID-19 has had a huge and negative effect on cancer treatment and research [4]. Patients with haematological cancers, and recipients of haematopoietic stem cell transplantation (HSCT), tend to be immunosuppressed by their disease or therapy, are generally older, often have multiple comorbidities and are at increased risk of COVID-19 infection [5, 6]. A retrospective analysis of 355 COVID-19 patients who died in Italy showed that 20% had active cancer, a study in HSCT patients found that 17% developed human coronavirus infections [7, 8]. Compromising treatment intensity or delaying treatment can significantly increase the risk of relapse and mortality. Continuing therapy with full dose intensity in these immune deficient patients can be the cause of high treatment related mortality in highly curable patients. The balancing act between curative treatments and high mortality was our major challenge during the ongoing pandemic.

Our hospital, like elsewhere, had to face this situation anticipating a rapid increase in cases and had to prepare accordingly with potential worst-case scenarios in mind. We share the experience of managing adults and children with haematological malignancies and bone marrow transplant at our centre in the city of Kolkata, which has been one of the declared hotspots of the COVID-19 pandemic in the country [9].

## Institutional challenges

At the outset, problems were created by logistic challenges which emerged due to the strict countrywide lockdown imposed by the government from 23 March 2020 [2]. This severely affected the supply chain of food essentials, drugs, laboratory reagents, radio-diagnostic agents, maintenance requirements and personal protective equipment (PPE) in the initial days. Infection prevention in a pandemic has a two-fold challenge of preventing infection in patients visiting the hospital, and among healthcare providers. Our hospital has an active infection control policy which primarily focuses on preventing hospital acquired infections. The challenge that a pandemic pose is that healthcare settings act as potential amplifiers in spreading an infection which is primarily community acquired. This hospital is exclusively for cancer (and related conditions) with most of its patients being severely immunocompromised. This increases the risk of transmission to care providers as well, as immunocompromised hosts are more likely to have a high viral load. The lack of adequate information in the initial phase, of the actual rate of spread in the community, was also affecting the ability to make policy decisions.

Maintaining safe distancing was a challenge with an outpatient division (OPD) footfall of 1,000–1,200 cancer patients per day, accompanied by attendants. A screening facility for patients and attendants to look for symptoms and fever was necessary and crowd control was essential especially since an average patient is accompanied by two attendants (at least). Protecting healthcare workers (median age at our institution: 27 years (18.8–70.4 years)) anxious by the fear of a yet unknown virus, maintaining staff morale, facilitating employee attendance and ensuring adequate coverage of services, were among many challenges the administration had to grapple with it. Exposing the entire workforce to this potential pandemic would have deleterious consequences, including maintenance of hospital services for patients. There were Government guidelines in this regard, by mid-April 2020 [10].

## Challenges in the management of COVID-19 patients with haematological cancers

The SARS-CoV-2 COVID-19 infection was expected to have deleterious outcomes among patients with haematological malignancies and hematopoietic stem cell transplant recipients. The Haematology unit registers 2,500 to 2,700, and the Paediatric division 500 to 600 new patients annually. The average daily in-patient strength of the two units, was 80 in the month before lockdown and was scaled down to less than 30. This was done to provide a ‘holding area’ for COVID suspects, isolation rooms for quarantining clinically stable COVID-19 infected patients and healthcare workers. The availability of high-dependency unit and intensive care unit beds were limited, and this was further complicated by the need to set aside a reserve number in the eventuality of a large outbreak. There was a major shortfall in volunteer blood donors coming to the hospital which led to a significant stretch in the resources of the blood bank and affected transfusion services across the hospital. In a Haematology service, decisions on intensive chemotherapy including admissions to a bone marrow transplant (BMT) facility also depend on blood product availability, and patient families had to provide donors as replacement blood banking. Being a not-for-profit private trust medical centre, we are dependent on funding agencies to provide for many of our patients undergoing curative treatments and who most often require financial assistance for intensive chemotherapy and supportive care. Offices of government agencies, non-governmental organisations and corporate social responsibility liaisons were either closed or constrained (with partial staffing), resulting in cash flow challenges initially, for patient funding and treatment delivery. Psychological comorbidities are common in any chronic medical illnesses including cancer. For many patients visiting our hospital, fear of cancer progression mattered more significantly than fear of COVID-19 [11]. The patients were seen to be additionally distressed by the constraints imposed by the lockdown, during this pandemic.

The therapy for COVID-19 has no standard of care and there are conflicting reports on the efficacy of drugs like hydroxychloroquine (HCQ) monotherapy (including prevention), HCQ combinations, Remdesivir, dexamethasone and many others in treatment [12–15]. Availability of generic drugs is not a compelling constraint here, as India has one of the largest production facilities for manufacturing generics [16]. Our challenge of formulating a treatment policy was complicated by the presence of multiple treatment guidelines and recommendations from several professional organisations, societies and Government authorities regarding COVID-19 infection, and the management of cancers during the COVID-19 pandemic [17].

## Institutional response

The problem posed by the COVID-19 pandemic was a challenge to the living memory of most personnel at our institution. The hospital administration reviewed best practices from published literature, cross consultation and experience from institutional memory [18]. A central command centre was established and made functional from 17 March 2020 in the hospital led by the chief executive and senior medical administrator. The command centre and COVID management team included all verticals of the administration (including Finance, Human resources, Quality, Customer care, engineering, staff health, security, etc.), infection control, laboratory services, imaging services, clinical staff, nursing and para-medical lead personnel, etc. In this unusual scenario, the clinical leadership consisting of all subspecialties of cancer care and diagnosis decided to organise themselves into a team led by a senior oncology physician, by 23 March 2020. The team’s mandate was to distil current information and draft (or modify) clinical pathways for the care of cancer patients and healthcare workers (HCW) who would be COVID-19 infection suspected or confirmed. This team was given the acronym TMC CLINCH team.

The COVID management team inclusive of the command centre, infection control team and CLINCH team formulated the administrative, diagnostics and infection control, and clinical pathway standard operating procedures (SOPs). Isolation facilities and levels of isolation were identified and earmarked, a physical space for the command centre was commissioned; and a hospital mock drill was conducted in 48 hours. Training of staff members began immediately once the draft administrative protocols were prepared by 27 March 2020. The clinical pathways and management protocols were formulated and issued in a week. Considering the dynamic situation in the community and rapidly changing directives from various agencies, the SOPs needed frequent modifications. They are currently in version 3 as this paper is being finalised.

The shortage of PPE in the initial phase of the outbreak took a couple of weeks to rectify. To overcome the shortage of the full complement of PPE, we had to ration its use to provide for staff at the highest risk of infection, e.g., nurses and doctors involved in the care of suspected or proven COVID-19 patients. Furthermore, the full complement of PPE was provided to staff involved in emergency, isolation and surgeries and other aerosol generating procedures. This challenge was mitigated once production of PPE was ramped up in the country. All patients and relatives were required to wear 3ply mask, N95 mask or cloth masks at all times while inside the hospital. Staff were divided into batches and rotational workforce practices were implemented on a weekly basis, to reduce the risk of a large number of staff falling sick at the same time and disrupt services and limit the use of PPE. At individual departmental levels, the departmental heads (or lead consultants) were advised and empowered to make necessary administrative and work-related changes in order to prepare for the scenario and a potential surge in the number of COVID-19 affected patients and HCW, considering the best and worst situations.

## Infection control

In addition to the existing infection control policy in the hospital, we set up protocols to guide patients and staff in preventing COVID-19 transmission. As early as January 2020, the infection control team initiated weekly monitoring of the COVID situation. Patients were screened at the time of entry into the hospital with a standard questionnaire and all deemed at risk for COVID-19 infection were shifted to a ‘suspect’ designated area, and later to isolation ward suites, for sample collection and testing. Testing kits validated by the National Institute of Virology and approved by Indian Council of Medical Research were procured to start RT-PCR for COVID-19 [19]. This included simultaneous detection of screening E-gene, and confirmatory S or RdRP gene as per the available kit. Patient with either of 1 gene positive were reported as ‘Indeterminate’, while Samples showing no PCR amplifications were tried two times before finally reported as ‘Inconclusive’. Patients with definitive symptoms or radiological findings as per the screening protocol, presenting to out-patient or emergency services also followed the same pathway. The isolation suites were re-organised to care for patients with intensive care support as well. Confirmed cases continued to be in isolation while others were shifted to the appropriate wards, cared for or discharged. The testing pathways were issued by 4 April 2020; as our centre was designated as a COVID-19 testing centre by the Indian Council of Medical Research (ICMR). Protocols for tracing and assessment of high-risk contacts were. A rapid response code to safely identify and transfer suspects to isolation areas was also implemented simultaneously. Testing criteria and protocols underwent periodic revisions based on directions and advisories from Government and professional health agencies. To ensure testing went on unhindered, staff were redeployed from different clinical and research areas and appraisal of testing kits and advance orders were fast-tracked. The research staff from the haematology departments also volunteered for these services.

## Clinical service and treatment policies

In order to understand the impact on services, we reviewed data on patients availing adult and paediatric haematology out-patient, inpatient, day-care and BMT services in our institution between March and May 2020 and compared it with the same time period in 2019, where applicable. The outpatient services saw a significant drop (47%), and the day care (12%) a less significant one. Inpatient bed-occupancy dropped by 50%. The paediatric services functioned unchanged initially. Transfusion medicine services saw a significant drop (*n* = 2,519 versus 1,877, 25%) in donations and blood products prepared, with consumption remaining more or less the same. Haematological malignancies were categorised into potentially curable, controllable and palliative. Potentially curable leukaemia and high-grade lymphoma continued to receive treatment with a curative intent using dose adjustments for age and myelosuppression. Growth factors as well as supportive care were advised to be used generously during therapy. In patients with early-stage Hodgkin’s lymphoma, the baseline and interim PET scan were utilised to minimise the number of chemotherapy cycles required. PET-directed therapy was used to avoid starting intensive BEACOPP regimen upfront for advanced disease. These regimens were used for patients with suboptimal response after the first two to three cycles of standard ABVD therapy. Radiation therapy was avoided and alternative regimens used to reduce repeated hospital visits. In case of relapsed leukaemia and lymphoma, outpatient-based salvage regimens such as GDP were used in place of more intensive ICE/ESHAP/DHAP therapies, where feasible. For indolent and controllable diseases, treatment in the outpatient clinic was encouraged with minimum OPD visits. Oral therapies replaced injectable management wherever possible. Maintenance therapy with rituximab in indolent lymphomas was postponed or temporarily withheld. Patients with multiple myeloma were advised to continue oral and subcutaneously administered therapy at home under supervision of house physicians, and hospital visits were reduced. This practise was in line with guidance from reputed professional societies and consensus statements [20–26]. All procedures such as high-dose chemotherapy and autologous hematopoietic stem cell procedure were conducted with due thought and postponed where feasible, after discussion within the multi-disciplinary team. Decisions on Allogeneic transplantation were similarly decided depending on the disease risk status, based on recommendations [21].

Among the patients tested or screened for COVID-19 from April 2020 to June 2020, infection was confirmed in 10 and indeterminate/inconclusive in 6, in the adult services. They COVID-19 positive patients were in various phases of remission induction therapies or initial evaluation phase for acute leukaemia (*n* = 3), aggressive non-Hodgkin lymphomas (*n* = 4), indolent lymphoma (*n* = 2) and multiple myeloma (*n* = 1). Two patients succumbed. Paediatric haematology section did not record any case positivity during this period, and had three indeterminate/inconclusive reports. As a part of a global effort, our departments are collaborating in the ASH research Collaborative initiative on COVID-19 in patients with haematological disorders and reporting cases to this registry [27]. Adult Haematology, Paediatric Haematology-Oncology and BMT clinical services have a staff strength of about 181 personnel. Across adult and paediatric haematology and BMT services, 52 members of staff were quarantined post exposure, and underwent COVID-19 testing prior to re-joining the clinical services. Two nurses working in the adult haematology service tested positive and asymptomatic, while all others tested negative. At the time of finalising this paper, the departments have reverted to working full hours.

## Patient follow-up and continuum of care

Patients continued to have difficulty in reaching the hospital though ambulances and private vehicles carrying patients to hospitals were allowed to ply. To overcome these difficulties at least partially, telephonic consultations, messenger services like WhatsApp® and emails were used for maintaining communication with patients. These services provided a channel for answering patient queries and providing treatment information. Online consultations between patients and clinicians in the institution have been predominantly informal in our institution as there were no clear laws regarding this form of patient interaction in India, for a long time. The medical council of India and ministry of health and family welfare, Government of India on 25 March 2020 issued legal sanction to online consultations by issuing guidelines for the same [28]. This pandemic, social distancing norms and legal sanction re-energised our institution to begin secure formal online and tele-consultations for patients from 18 June 2020.

## Academic and research activities

The social distancing norms within the hospital meant cancelling most of our formal academic activities, including multidisciplinary meetings. All scheduled conferences planned to be conducted at the hospital were cancelled. Staff involved in research activities were advised to work from home, and recruitment of patients to clinical trials dropped consistent with the drop in hospital footfall. From no recruitments in the first month of the lockdown, trial screenings restarted from the second month and have recorded recruitments at 100% pre-lockdown levels. Active biobanking was withheld. Interactions with trial sponsors and research organisations shifted to internet web-based platforms. A revised online format of institutional review board meetings was notified and conducted at the time of manuscript preparation, and the departments had presented three studies on the new online platform. By mid-May 2020, academic activities and multi-disciplinary meetings in the haematology department were restarted through a popular internet platform for web-based team activities. This has been running successfully, as this paper was being finalised. We have two accredited and three institutional post graduate training programmes in adult and paediatric haematology which undergo periodic evaluations. As these are time bound fellowship programmes, we did not create disruption in the assessment schedules and conducted these assessments while practicing social distancing norms.

## Conclusion

Faced with the threat of COVID-19, our administration first made local changes, put their acts into practice at a record speed to provide all logistics for the functioning of the hospital, staff protection and most importantly patient care. The first step was to shed our fear which initially gripped the staff. Care of cancer patients with COVID-19 needed special nursing and medical care, and constant revisions of the CLINCH document which provided information on PPE usage, screening of patients and their relatives, isolation, testing of suspects, management of the primary malignancy as well as SARS-CoV-2 in patients, treatment of the staff affected by COVID-19 and managing the dead. The care of haematological malignancies at our centre has been altered to mitigate the risks of COVID-19 in our vulnerable patient population and also to balance resource utilisation at this economically difficult time. Common themes include to continue the curative including allogenic stem cell transplants with all supportive care, use oral and outpatient medications in controllable disease, defer treatments known to be associated with high risk of viral infections in maintenance phases and increase Telemedicine facilities for patients on ‘wait and watch’ policy for low-grade malignancies as well as survivors on follow up, wherever possible our recommendations were evidence based and represented a consensus opinion.

## Conflicts of interest

None of the authors have any significant conflicts of interest regarding this work.

## Funding disclosure

None.

## References

[1] World Health Organization (2020). WHO Director-General’s Opening Remarks at the Media briefing on COVID-19—11 March 2020.

[2] Ministry of Home Affairs GoI (2020). Government of India Issues Orders Prescribing Lockdown for Containment of COVID-19 Epidemic in the Country.

[3] World Health Organization (2020). India situation report—25, Novel Coronavirus Disease (COVID-19). https://www.who.int/docs/default-source/wrindia/situation-report/india-situation-report-25.pdf?sfvrsn=8269893f_2.

[4] Saini KS, de Las Heras B, de Castro J (2020). Effect of the COVID-19 pandemic on cancer treatment and research. Lancet Haematol.

[5] Zhang L, Zhu F, Xie L (2020). Clinical characteristics of COVID-19-infected cancer patients: a retrospective case study in three hospitals within Wuhan, China. Ann Oncol.

[6] You B, Ravaud A, Canivet A (2020). The official French guidelines to protect patients with cancer against SARS-CoV-2 infection. Lancet Oncol.

[7] Onder G, Rezza G, Brusaferro S (2020). Case-Fatality Rate and Characteristics of Patients Dying in Relation to COVID-19 in Italy. JAMA.

[8] Eichenberger EM, Soave R, Zappetti D (2019). Incidence, significance, and persistence of human coronavirus infection in hematopoietic stem cell transplant recipients. Bone Marrow Transplant.

[9] Ministry of Home Affairs GoI (2020). Directions to implement the guidelines/ consolidated revised guidelines on lockdown and other measures for the containement of spread of COVID-19 in the country, No. 40-10/2020-DM-I(A). https://www.mha.gov.in/sites/default/files/WestBengal_20042020.pdf.

[10] Ministry of Health and Family Welfare Government of India (2020). Measures undertaken to ensure safety of health workers drafted for COVID19 Services, D.O.No.7-20015/127/2019-ME.I(Pt.I). https://www.mohfw.gov.in/pdf/MeasuresUndertakenToEnsureSafetyOfHealthWorkersDraftedForCOVID19Services.pdf.

[11] Ghosh J, Ganguly S, Mondal D (2020). Perspective of oncology patients during COVID-19 pandemic: a prospective observational study from India. JCO Glob Oncol.

[12] Mehra MR, Desai SS, Kuy S (2020). Retraction: cardiovascular disease, drug therapy, and mortality in Covid-19. N Engl J Med.

[13] The Lancet E (2020). Expression of concern: hydroxychloroquine or chloroquine with or without a macrolide for treatment of COVID-19: a multinational registry analysis. Lancet.

[14] Beigel JH, Tomashek KM, Dodd LE (2020). Remdesivir for the treatment of Covid-19—preliminary report. N Engl J Med.

[15] Horby P, Lim WS, Emberson JR (2020). Dexamethasone in hospitalized patients with Covid-19—preliminary report. N Engl J Med.

[16] Ministry of Health and Family Welfare GoI (2020). Updates on COVID-19.

[17] Xu X, Ong YK, Wang Y (2020). Role of adjunctive treatment strategies in COVID-19 and a review of international and national clinical guidelines. Mil Med Res.

[18] Pramesh CS, Badwe RA (2020). Cancer management in India during Covid-19. N Engl J Med.

[19] Ministry of Health and Family Welfare Government of India Notification of ICMR guidelines for COVID19 testing in private laboratories in India. F.No.z.28015/23/2020-EMR.

[20] American Society of Hematology (2020). Resources for Clinicians. https://www.hematology.org/covid-19.

[21] Damodar S, Radhakrishnan VS, John JM P, Jain R, Melinkeri S, Easow J, Srivastava A (2020). HSCT Guidelines for transplant practises during COVID-19 pandemic in India. Blood Cell Therapy.

[22] European Society for Medical Oncology (2020). Cancer patient management during the COVID-19 pandemic: ESMO. https://www.esmo.org/guidelines/cancer-patient-management-during-the-covid-19-pandemic?page=2.

[23] Alpana Waghmare on behalf of ASTCT Infectious Disease Special Interest Group (2020). ASTCT interim patient guidelines for COVID-19 management in Hematopoietic Cell Transplant and Cellular therapy patients, Version 1.3. https://www.astct.org/viewdocument/astct-interim-patient-guidelines-ap?CommunityKey=d3949d84-3440-45f4-8142-90ea05adb0e5&tab=librarydocuments&LibraryFolderKey=1822c414-8535-4510-bb3d-13958fea4b85&DefaultView=folder.

[24] Ljungman P, Mikulska M, de la Camara R (2020). The challenge of COVID-19 and hematopoietic cell transplantation; EBMT recommendations for management of hematopoietic cell transplant recipients, their donors, and patients undergoing CAR T-cell therapy. Bone Marrow Transpl.

[25] St. Jude Global The global COVID-19 observatory and resource center for childhood cancer: St. Jude Global and SIOP. https://global.stjude.org/en-us/global-covid-19-observatory-and-resource-center-for-childhood-cancer.html.

[26] Children’s Cancer and Leukaemia Group (2020). COVID-19 guidance for children and young people with cancer undergoing treatment. https://www.cclg.org.uk/write/MediaUploads/About%20CCLG/CCLG_COVID_Guidance_17.06.20.pdf.

[27] ASH Research Collaborative (2020). COVID-19 Registry: American Society of Hematology. https://www.ashresearchcollaborative.org/s/covid-19-registry.

[28] Board of Governors in supersession of Medical Council of India (2020). Telemedicine Practise Guidelines, enabling registered medical practitioners to provide healthcare using telemedicine: Ministry of Health and Family Welfare. https://www.mohfw.gov.in/pdf/Telemedicine.pdf.

